# Impact of Novel Varietal and Regional Differences on Cotton Fiber Quality Characteristics

**DOI:** 10.3390/ma15093242

**Published:** 2022-04-30

**Authors:** Azmat Hussain, Muhammad Sajid, Danish Iqbal, Muhammad Ilyas Sarwar, Assad Farooq, Amna Siddique, Muhammad Qamar Khan, Ick-Soo Kim

**Affiliations:** 1 College of Textile Engineering, Bahauddin Zakariya University, Multan 60800, Pakistan; azmathussain@bzu.edu.pk (A.H.); msajid@bzu.edu.pk (M.S.); 2 Shandong Center for Engineered Nonwovens, Industrial Research Institute of Nonwovens & Technical Textiles, College of Textiles & Clothing, Qingdao University, Qingdao 266071, China; danish.iqbal@ymail.com; 3 Central Cotton Research Institute, Multan 60000, Pakistan; mianilyas222@yahoo.com; 4 Department of Fiber and Textile Technology, University of Agriculture, Faisalabad 38000, Pakistan; assadfarooq@uaf.edu.pk; 5 School of Engineering and Technology, National Textile University, Faisalabad 37610, Pakistan; 6 Nanotechnology Research Group, Department of Textile and Clothing, Faculty of Engineering and Technology, National Textile University Karachi Campus, Industrial Area Korangi, Karachi 74900, Pakistan; 7 Division of Frontier Fiber, Institute of Fiber Engineering, Interdisciplinary Cluster for Cutting Edge Research (ICCER), Faculty of Textile Sciences, Shinshu University, Nagano 3868567, Japan

**Keywords:** cotton variety, fiber characteristics, cotton regions, climate effect, temperature effect

## Abstract

Modernization and the global fashion market demand continuous improvements in upland cotton cultivars (*Gossypium hirustum* L.) to meet these improved fiber characteristics (fiber length, fiber strength, micronaire) requirements. Researchers have centered their efforts on improved fiber quality; however, the efforts are not immediately supporting the textile sector. The daily mean and temperature amplitude fluctuation affect cotton yield and fiber characteristics. This study analyzed four newly developed cotton varieties in two cotton regions for fiber characteristics’ variations. It was observed that cotton fiber quality characteristics (fiber length, uniformity, strength, and micronaire) are impacted in diverse ways. Fiber quality is mainly affected by the genotype and environmental conditions, e.g., weather conditions, irrigation management, fertilization, and cultural practices. The Khanewal region had shown better fiber characteristics than the Multan region, whereas cotton variety CIM-785 had better fiber characteristics in both regions.

## 1. Introduction

Cotton (*Gossypium hirsutum* L.) is a plant fiber crop with a long history of textile usage due to its superior comfort over synthetic fibers. In addition, the natural cellulose fiber, cotton, has factually been the most used material in medical products (stitches, absorbent pads, dressings, and bandages) and continues to account for significant volumes of the absorbent and dressing products used worldwide. Cotton varieties in Punjab are certified based on fiber quality norms such as fiber strength of 25 g/tex and above, length of 28 mm, and micronaire of 3.8 to 4.9 [1]. Producers are offered a variety of upland cotton varieties each year. Plant type, maturity, fiber qualities, added-value features (e.g., insect and herbicide resistance transgenes), yield, and environmental adaptability characterize these types. Both governmental and private groups undertake multilocation cultivar trials to analyze plant and fiber performance to aid growers. Cotton fibers that are stronger, longer, finer, and more consistent are needed by contemporary textile manufacturers.

Cotton fiber growers and processors are concerned with the fiber’s strength, length, fineness, color, and trash content. Compared to traditional ring spinning technologies, the fiber quality standards for current high-speed yarn production procedures at higher spinning speeds [2,3]. As a result of this issue, processors are compelled to employ higher-strength cotton fiber in their yarn production.

Textile spinning mills are continually looking for methods to improve the quality-to-cost ratio. They must also establish processing ratios obtained by combining fibers from several bales with similar qualities. Cotton growers may see differences in the qualities of fibers, such as color variation, but no device can predict quality parameters as they develop in the boll [4,5]. There is no reference source, database, or model to foresee how atmospheric and genetic changes can be adapted to the cotton plant’s cultivating conditions. It is crucial to understand how climate and soil affect cotton cultivars.

Cotton quality may be determined by seed or fiber qualities. However, it is most typically connected to fiber attributes. Cotton fiber quality has improved due to increased global competition in production and use of cotton fiber and technical advancements in yarn manufacture. Genetics, crop management, and postharvest distribution may help improve cotton fiber quality. It is critical to understand the impact of fiber qualities on processing and their heredity, interactions, and environmental factors to develop improvement solutions. For yarn production systems, breeding to improve fiber quality has traditionally focused on improving measurements of the longest fibers or fiber strength. Variability in fiber qualities is caused by the environment, which makes it challenging to enhance them by breeding or biotechnological means. Because fiber processing is hindered by variability in fiber qualities, future breeding and biotechnological methods should simultaneously enhance fiber properties and lower variance [6]. 

The purpose of the current research project was to investigate regional and varietal variations in fiber quality features and their influence on yarn parameters in the final product. Currently, farmers produce cotton with high-yielding varieties without considering the fiber characteristics, and the textile mills have to import cotton for better fiber properties. The main objective of this study is to explore the varietal behavior of newly developed cotton varieties concerning environmental conditions.

## 2. Materials and Methods

The present research entitled “impact of novel varietal and regional differences on cotton fiber quality and their implication to yarn properties” was initiated in the College of Textile Engineering, Bahauddin Zakariya University, Multan, in the year 2021. The four cotton cultivars from Central Cotton Research Institute, Multan were selected with two cotton regions for this experiment. A significant reduction in flower and boll retention was observed at high temperatures (>36 °C), which ultimately resulted in a severe decrease in seed cotton yield and vice versa. The seed cotton was picked and ginned on a miniature ginning machine (Chaudhary Engineering Works, Multan, Pakistan). The following sections provide information on the materials and procedures utilized to test the different quality features of raw cotton. The following variables were selected for this research work, and all are given in Table 1.

### 2.1. Analysis of Data

A least significant difference (LSD) test is used in the context of the analysis of variance. The LSD calculates the smallest significance between two means as if a test had been run on those two means (as opposed to all of the groups together). Any difference greater than the LSD is considered a significant result.

The formula for the least significant difference is:

$$\mathrm{LSD}=t._{0.05},\;\mathrm{DFw}\;\times \;\sqrt{MSw\left(1/n+1/n\right)}\;$$

where:

**t** = critical value from the t-distribution table;

**MSw** = mean square within, obtained from the results of your ANOVA test;

**DF_w_** is the degrees of freedom within groups from the ANOVA table;

**n** = sample size.

### 2.2. Fiber Physical Characteristics

High Volume Instrument (HVI) 1000 is used for fiber physical characteristics. The Length/Strength Module measures two samples simultaneously; places approximately 8 to 10 g of fiber in each sample bucket; and it automatically prepares the comb from the material in each bucket. The combs slide along the comb track until the first one is positioned in front of the brusher. As soon as the comb is in place, the brusher automatically removes loose fiber from the beard while simultaneously cleaning and aligning the remaining fibers. It is then moved along the comb track to the lens and jaw system, where the fiber beard is scanned from base to tip for measuring length, uniformity, and short fiber index. Length uniformity is the ratio between the mean length and upper half mean length of the fibers and is expressed as a percentage. Micronaire is the measure of fiber fineness and maturity. An airflow instrument measures the air permeability of weighing between 8.5 to 11.5 g of cotton fibers compressed to a fixed volume.

The testing technique shall be followed as specified by the ASTM committee (D-5867-12) [7]. The testing will be carried out under standard laboratory conditions, which include a relative humidity of (65 ± 2)% and (20 ± 2) °C temperature ASTM standard method (D-1776) [8].

### 2.3. Fiber Mechanical Characteristics

High Volume Instrument (HVI) 1000 is used for fiber mechanical characteristics. The Length/Strength Module measures two samples simultaneously; places approximately 8 to 10 g of fiber in each sample bucket; and it automatically prepares the comb from the ma-terial in each bucket. Fiber strength and elongation are measured by breaking the tapered beards using 3.2 mm (1/8 inch) clamp spacing. The testing technique shall be followed as specified by the ASTM committee (D-5867-12) [7]. The testing will be carried out under standard laboratory conditions, which include a relative humidity of (65 ± 2)% relative humidity and (20 ± 2) °C temperature ASTM standard method (D-1776) [8].

### 2.4. Fiber Chemical Characteristics

#### Cellulose, Wax Content, and Ash Content

In cotton fibers, crystallites of native cellulose are composed of molecules with their reducing groups at one end of the crystal, described as similar packing. Since the crystallites are generally aligned with the fiber axis, one might describe the crystallite as oriented with the reducing end of the crystal towards the growth tip of cotton fiber. Cellulose, wax, and ash content were estimated by the method as prescribed by A.O.A.C. (1990) [9]. A weighted sample of one gram oven-dried cotton was digested in 200 mL, in addition to 1.25% H_2_SO_4_ for thirty minutes with gentle boiling on an electric heater up to 80°C using a condenser for maintaining a constant volume of solution. The sample was filtered through a thick linen cloth and washed with distilled water till it was free from acid. After that, the sample was digested in a 1.25 percent NaOH solution. The digested sample was filtered and thoroughly washed to the point that it gave no pink color with a phenolphthalein indicator. Finally, washing was done with 95 (%) ethanol. The samples were then dried at 70 °C to a constant weight and ash in an automatically controlled muffle furnace at 450 to 500 °C for not more than 30 min. The cellulose content of cotton was calculated using the following mathematical expression:

$$\mathrm{Cellulose}\;\mathrm{Percentage}=\frac{Oven\;dry\;weight\;after\;treatment-Ash\;Weight}{Dry\;sample\;total\;weight}\times 100\;$$

For wax content, a five-gram sample of raw cotton was placed in thimble filter paper in a Soxhlet extractor and the solvent “Light Petroleum Ether” poured through the condenser until the siphon operated. A further 10 to 20 mL solvent was added, and extraction was started by turning the water bath on, and thermo-state was fixed in the range of 85 °C to 95 °C. The extraction was done for three hours while solvent was siphoned at least six times per hour. The apparatus was disconnected and the extract was evaporated carefully to dryness, and the extract was weighted after oven drying. The amount of wax was represented based on the original cotton weight.

For ash content, a weighed amount of the material previously dried at 110 °C was placed in a muffle furnace at about 500 ± 50 °C for 30 min. The residual ash was weighed in a close vessel. Covered crucibles were used to put the sample in them. The amount of ash was calculated based on the original cotton weight.

## 3. Results and Discussion

### 3.1. Fiber Length (mm)

Fiber length is generally the object of trait development efforts in cotton since it is an important fiber quality feature in spinning technology, and it is genetically determined [10]. The results of the analysis of variance of the data related to fiber length are shown in Table 2, which demonstrates that the influence of cotton varieties (V) and cotton regions (R), as well as interactions, were highly significant in this study. The least significant difference (LSD) test is used in the context of the analysis of variance, and the comparison of individual treatment means for different varieties presented in Table 3 showed that the mean values of fiber length for V_1_, V_2_, V_3_, and V_4_ were 28.44, 27.91, 28.69, and 27.31 mm, respectively. Following the analysis, it was discovered that the fiber length values for various types are significantly varied from one another. The results are supported from Hameed [11], and the staple length of several Pakistani cotton cultivars ranges between 26.92 and 29.72 mm, which is extremely similar to the data obtained by this research. The length of the fiber was also highlighted by Hsieh and Hu [12], who said that it was influenced by both varietals and growth variables (environmental and developmental factors). 

The least significant difference (LSD) test and the comparison of individual treatment means for both regions shown in Table 3. The mean values of staple length for R_1_ and R_2_ were 27.60 and 28.48 mm, respectively. Fiber length values for distinct locations were shown to be considerably varied from one another, based on the findings of this study. These findings are supported by earlier research given by the author [13]. NIAB-111 had a mean span length of 29.64 and 30.00 mm, according to the researchers. In a similar vein, Nazar and Iftikhar [14] discovered that the staple length of several Pakistani cotton cultivars ranges from 26.92 to 29.72 mm, depending on the variety. In addition, El Mogahzy and Gowayed [15] indicated that fiber length was an essential property in assessing cotton quality, which was crucial to spinners since it was strongly connected to processability and the quality of yarn produced.

**Figure 1:**
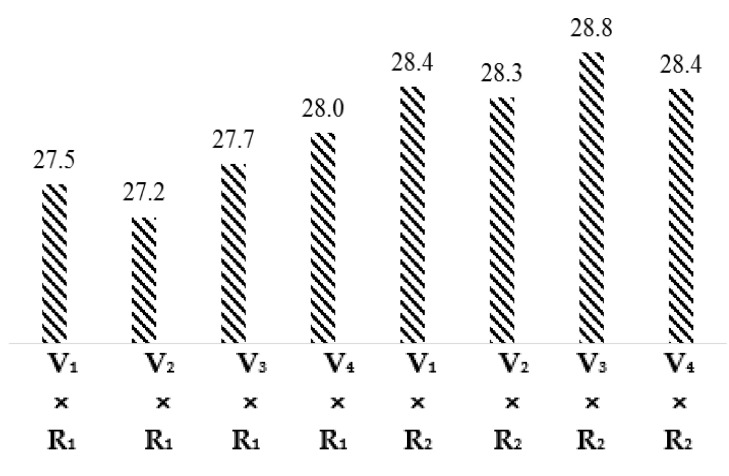
Graphical Representation interaction means for Fiber Length (mm).

The least significant difference (LSD) test and the comparison of interaction means for varieties and regions presented in Figure 1 show that the fiber length values of interactions V_1_ × R_1_, V_2_ × R_1_, V_3_ × R_1_, V_4_ × R_1_, V_1_ × R_2_, V_2_ × R_2_, V_3_ × R_2_, and V_4_ × R_2_ are 27.5, 27.2, 27.7, 28.0, 28.4, 28.3, 28.8, and 28.4 mm, respectively. The fiber length of all cotton varieties in the Multan region was lower than the Khanewal region. This is due to the environmental effect and soil structure differentiation. The night temperature of Khanewal is lesser than in the Multan region during the cotton season.

### 3.2. Fiber Strength (g/tex)

Fiber strength is the link between the busted strength of the fiber and the assembly of the fiber that has been broken. Knowledge of yarn strength within a cotton cultivar or across cultivars may be beneficial in assisting with cultivar selection [16]. Table 4 demonstrated that the influence of cotton varieties (V) and cotton regions (R) and interactions were highly significant when it came to fiber strength measurements. The least significant difference (LSD) test is used in the context of the analysis of variance, and the comparison of individual treatment means for various cotton varieties given in Table 5 presented that the mean values of fiber strength for V_1_, V_2_, V_3_, and V_4_ were, 28.88, 28.90, 30.49, and 28.38 g/tex, respectively. The findings revealed that the fiber strength values for various types are statistically significantly different. These findings are supported by a research study conducted by Ahmad [17], who said that the fiber bundle strength of several cotton types in Pakistan ranged from 21.80 to 28.42 g/tex for distinct kinds. As previously indicated, Cui and Price [18] found that yarn strength was substantially described by fiber strength.

The least significant difference (LSD) test and the comparison of individual treatment means for both regions given in Table 5 indicate that the mean values of fiber strength for R_1_ and R_2_ were 28.55 and 29.76 g/tex, respectively. The study discovered that the fiber strength values obtained from various places differed greatly from one another. These findings are supported by a study conducted by Hsieh and Hu [12], who found that poorer tensile characteristics of fibers from mature bolls might be caused by exposure to diverse settings, such as prolonged time on the plant or exposure to more significant amounts of moisture and heat.

**Figure 2:**
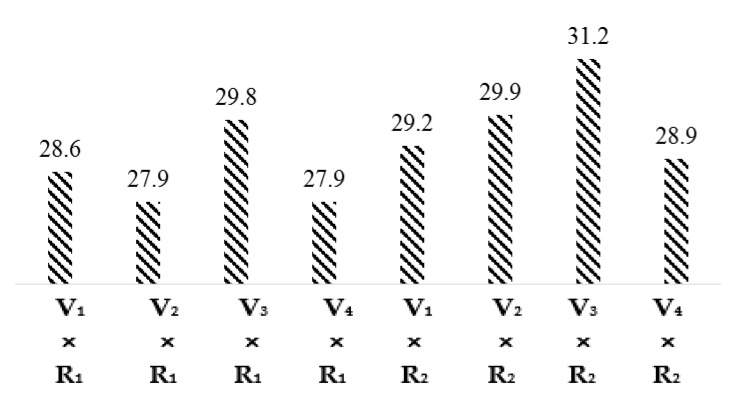
Graphical representation interaction means for Fiber Strength (g/tex).

The least significant difference (LSD) test and the comparison of interaction means for varieties and regions given in Figure 2 indicated that the fiber strength values of interactions V_1_ × R_1_, V_2_ × R_1_, V_3_ × R_1_, V_4_ × R_1_, V_1_ × R_2_, V_2_ × R_2_, V_3_ × R_2_, V_4_ × R_2_ are 28.6, 27.9, 29.8, 27.9, 29.2, 29.9, 31.2, and 28.9 g/tex, respectively. Fiber strength of all cotton varieties in the Multan region was lower than the Khanewal region due to high night temperature in Multan. 

### 3.3. Micronaire

Fiber micronaire is a key quality characteristic since it is an indirect indicator of fiber linear density (fineness) and maturation and is impacted by crop supply and assimilation partitioning to cotton fruit [19]. The results of the analysis of variance of the data related to micronaire are shown in Table 6, which demonstrates that the influence of cotton varieties (V) and cotton regions (R) and interactions were highly significant. The least significant difference (LSD) test is used in the context of the analysis of variance, and the comparison of individual treatment means for various cotton varieties given in Table 7, which presented that the mean values of micronaire for V_1_, V_2_, V_3_, and V_4_ were, 4.57, 4.58, 4.96, and 4.14, respectively. The research study conducted by Liu et. al. [20] supports these findings, and the substantial variability in single fiber qualities within a single variety shows that growing circumstances and development have a very strong impact on the fiber’s properties. Micronaire was the most critical cotton fiber quality in determining product quality and market value of yarn. In contrast, overall differences in single fiber tensile qualities were found to be more closely connected with seed location in the boll and less closely associated with fiber length. Brushwood [21] noted that fiber micronaire had a significant impact on the amount of noncellulosic ethanol extractable, wax, fiber ash residues, and potassium concentration on the fibers and the amount of friction created during the carding sliver processing.

The least significant difference (LSD) test and the comparison of individual treatment means for both climatic regions given in Table 7 presented that the mean values of micronaire for R_1_ and R_2_ were 4.84 and 4.29, respectively. The findings demonstrated that the micronaire values for both locations differed considerably in a statistically meaningful way. Between 4.29 and 4.84 was a suitable range of micronaire for the chosen cultivars.

**Figure 3:**
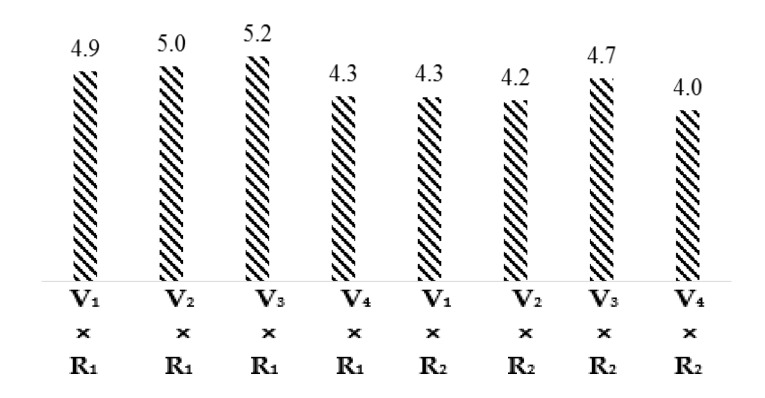
Graphical Representation interaction means for Micronaire.

The least significant difference (LSD) test and the comparison of interaction means for cotton varieties and climatic regions given in Figure 3 demonstrated that the micronaire values of interactions V_1_ × R_1_, V_2_ × R_1_, V_3_ × R_1_, V_4_ × R_1_, V_1_ × R_2_, V_2_ × R_2_, V_3_ × R_2_, and V_4_ × R_2_ are 4.9, 5.0, 5.2, 4.3, 4.3, 4.2, 4.7, and 4.0, respectively. Micronaire of all cotton varieties in the Multan region was higher than in the Khanewal region because of the higher temperature in Multan regions, as previous researchers have found that linear responses of micronaire to temperature have been reported [22,23].

### 3.4. Cellulose (%)

The results of the analysis of the variance of the data related to cellulose (%) are shown in Table 8, which demonstrates that the influence of cotton varieties (V) and cotton regions (R) and interactions were highly significant. The least significant difference (LSD) test is used in the context of the analysis of variance, and the comparison of individual treatment means for various cotton varieties given in Table 9, which presented that the mean values of cellulose (%) for V_1_, V_2_, V_3_, and V_4_ were 89.25, 90.71, 95.10, and 87.75, respectively. These results get support from the research study by Umar [24], which stated the range for cellulose content for some Pakistani medium staple cotton as 86.4 to 89.3 percent. In addition, Montalvo [25] stated that cotton with a much greater genetic diversity was being developed, and a more excellent range of both fiber perimeter and wall thickness, and their combinations, was probable. Wakelyn et al. [26] stated that there was 88.0 to 96.5% of cellulose in mature cotton fiber.

The least significant difference (LSD) test and the comparison of individual treatment means for both climatic regions given in Table 9 presented that the mean values of cellulose (%) for R_1_ and R_2_ were 89.85 and 91.55, respectively. The findings demonstrated that the cellulose (%) for both locations differed considerably from one another in a statistically meaningful way. These results support the research study by Wang et al. [27] which stated that fiber strength was positively related to the maximal rate of cellulose increase, and this difference is likely caused by temperature variation.

**Figure 4:**
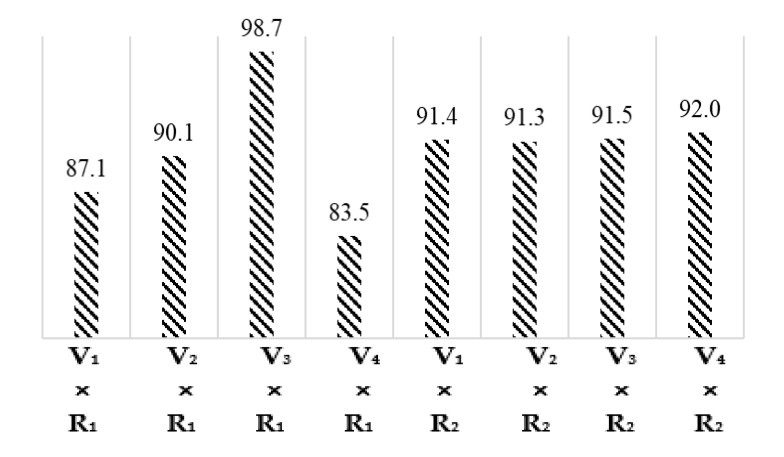
Graphical representation of interaction means for Cellulose (%).

The least significant difference (LSD) test and the comparison of interaction means for cotton varieties and climatic regions given in Figure 4 demonstrated the micronaire values of interactions V_1_ × R_1_, V_2_ × R_1_, V_3_ × R_1_, V_4_ × R_1_, V_1_ × R_2_, V_2_ × R_2_, V_3_ × R_2_, V_4_ × R_2_ are 87.1, 90.1, 98.7, 83.5, 91.4, 91.3, 91.5, and 92.0, respectively. Cellulose (%) of all cotton varieties in the Multan region was higher than in the Khanewal region.

### 3.5. Wax (%)

Cotton fibre total wax has been proven to operate as a lubricant during textile processing, and also has been shown to be adversely linked with key quality characteristics. The fully grown cotton cuticle is constituted mostly of two types of lipids: free waxes and cutin [28]. The results of the analysis of the variance of the data related to wax (%) are shown in Table 10, which demonstrates that the influence of cotton varieties (V) and cotton regions (R) and interactions were highly significant. The least significant difference (LSD) test is used in the context of the analysis of variance, and the comparison of individual treatment means for various cotton varieties given in Table 11, which presented that the mean values of wax (%) for V_1_, V_2_, V_3_, and V_4_ were, 0.59, 0.63, 0.60, and 0.69, respectively. These results get support from the research study by Brushwood [29] which stated that fiber micronaire increased when the concentrations of waxes decreased.

The least significant difference (LSD) test and the comparison of individual treatment means for both climatic regions given in Table 11 presented that the mean values of wax (%) for R_1_ and R_2_ were 0.64 and 0.62, respectively. The findings demonstrated that the wax (%) for both locations differed considerably from one another in a statistically meaningful way. These results support the research study by Hussain [30], which concluded that wax content correlates positively with fiber staple length, CLSP value, elongation, and RKM value while being negatively correlated with thin places of yarn.

**Figure 5:**
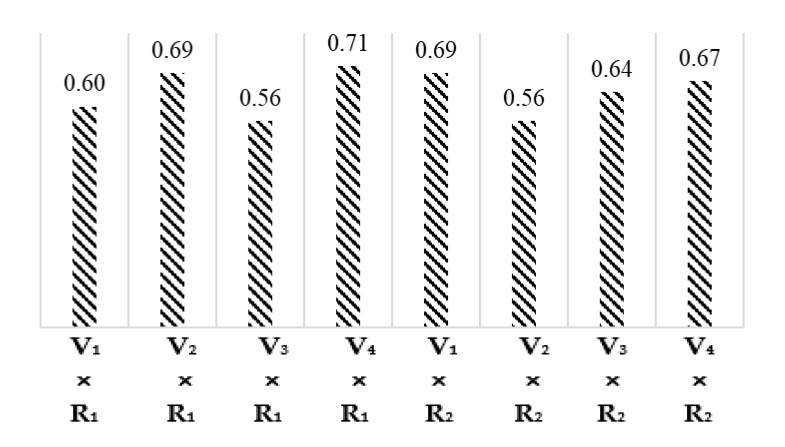
Graphical representation interaction means for Wax (%).

The least significant difference (LSD) test and the comparison of interaction means for cotton varieties and climatic regions given in Figure 5 demonstrated that the wax (%) values of interactions V_1_ × R_1_, V_2_ × R_1_, V_3_ × R_1_, V_4_ × R_1_, V_1_ × R_2_, V_2_ × R_2_, V_3_ × R_2_, and V_4_ × R_2_ are 0.60, 0.69, 0.56, 0.71, 0.69, 0.56, 0.64, and 0.67, respectively.

### 3.6. Ash (%)

The results of the analysis of the variance of the data related to ash (%) are shown in Table 12, which demonstrates that the influence of cotton varieties (V) and cotton regions (R) and interactions were highly significant. The least significant difference (LSD) test is used in the context of the analysis of variance, and the comparison of individual treatment means for various cotton varieties given in Table 13, which presented that the mean values of ash (%) for V_1_, V_2_, V_3_, and V_4_ were, 1.44, 1.37, 1.46, and 1.41, respectively. These results support the research study by Naeem [31], which reported that ash contents for different varieties range between 1.397 to 1.526 percent.

The least significant difference (LSD) test and the comparison of individual treatment means for both climatic regions given in Table 13 presented that the mean values of ash (%) for R_1_ and R_2_ were 1.43 and 1.41, respectively. The findings demonstrated that the ash (%) for both locations differed considerably in a statistically meaningful way. These results support the research study by Brushwood (2002) [32] that cotton from areas where open bolls normally were exposed to too little or no moisture generally had higher metal contents and ash residues.

**Figure 6:**
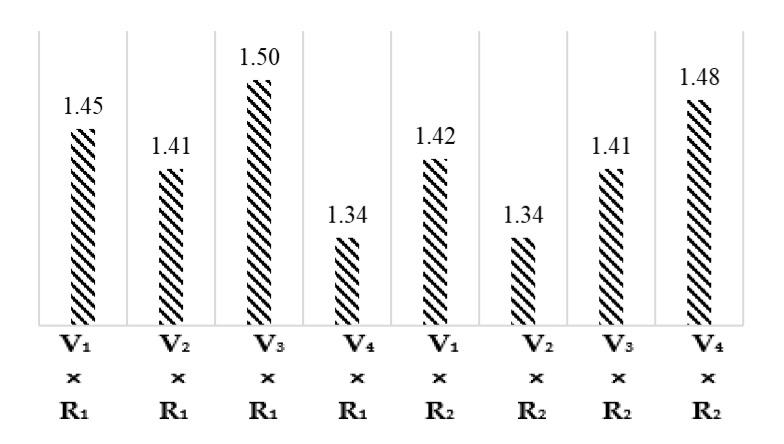
Graphical representation interaction means for Ash (%).

The least significant difference (LSD) test and the comparison of interaction means for cotton varieties and climatic regions given in Figure 6 demonstrated that the ash (%) values of interactions V_1_ × R_1_, V_2_ × R_1_, V_3_ × R_1_, V_4_ × R_1_, V_1_ × R_2_, V_2_ × R_2_, V_3_ × R_2_, and V_4_ × R_2_ are 1.45, 1.41, 1.50, 1.34, 1.42, 1.34, 1.41, and 1.48%, respectively.

### 3.7. Correlation Analysis

Correlation coefficients among the different characteristics of cotton fiber are presented in Table 14. The table shows that the fiber strength was found to be highly correlated with fiber length, cellulose (%), and wax (%) with values of 0.683, 0.539, and −0.578, respectively. Similarly, micronaire was correlated with fiber length (−0.675) and ash (%) (0.433), whereas wax (%) was also correlated with cellulose (%) with a value of 0.529.

## 4. Conclusions

Production regions affect cotton quality characteristics, which means that areas may be chosen, so cultivars are most effectively differentiated in cotton fiber quality. Aside from that, the growing zones must be selected to enhance fiber characteristics to achieve the best possible level of fiber quality. Cotton fiber quality characteristics (both physical and chemical) are impacted by environmental fluctuations, which means that ranking cultivars according to environmental variations are critical for improving cotton fiber quality. The environment affected the speed of fiber elongation even though genetic factors played a significant role. Fiber parameters that are important for the textile industry were altered by temperature. The decline in fiber length at high temperatures was more significant than at low temperatures. Fiber strength increased linearly with temperature. Micronaire and fiber uniformity showed quadratic trends with temperature. Cotton cultivar CIM-785 displayed better fiber characteristics in both regions. All varieties showed better performance in the Khanewal region than in the Multan region, implying that the Khanewal region has a favorable climate for cotton fiber characteristics.

## Figures and Tables

**Table 1:** Selected variables.

Cotton Varieties V	Cotton Regions R
V_1_ = CIM-663	
V_2_ = CIM-678	R_1_ = Multan
V_3_ = CIM-785	R_2_ = Khanewal
V_4_ = Cyto-535	

**Table 2:** ANOVA table for fiber length (mm).

Source	DF	SS	MS	F	P
V	3.0000	0.8667	0.2889	408.2100	0.0000 **
R	1.0000	4.6376	4.6376	6552.7100	0.0000 **
V × R	3.0000	0.4743	0.1581	223.3900	0.0000 **
Error	14.0000	0.0099	0.0007		
Total	21.0000	5.9885			

** = Highly significant, where DF = Degree of freedom, SS = Some of square, MS = Mean square, F = F-statistic, P = *p*-value.

**Table 3:** Individual mean comparison values for fiber length (mm).

Cotton Varieties (V)	Cotton Regions (R)
V_1_ = 27.97 B	
V_2_ = 27.76 C	R_1_ = 27.60 B
V_3_ = 28.23 A	R_2_ = 28.48 A
V_4_ = 28.21 A	

Mean values with various characters varied by 0.05 probability.

**Table 4:** ANOVA table for fiber strength (g/tex).

Source	DF	SS	MS	F	P
V	3.0000	15.1437	5.0479	1628.3548	0.0000 **
R	1.0000	8.9915	8.9915	2900.4838	0.0000 **
V × R	3.0000	1.6967	0.5656	182.4516	0.0000 **
Error	14.0000	0.0437	0.0031		
Total	21.0000	25.8756			

** = Highly significant.

**Table 5:** Individual mean values comparison for Fiber Strength (g/tex).

Cotton Varieties (V)	Cotton Regions (R)
V_1_ = 28.88 B	
V_2_ = 28.90 B	R_1_ = 28.55 B
V_3_ = 30.49 A	R_2_ = 29.76 A
V_4_ = 28.38 C	

Mean values with various characters varied by 0.05 probability.

**Table 6:** ANOVA table for Micronaire.

Source	DF	SS	MS	F	P
V	3.0000	2.0400	0.6800	1398.2500	0.0000 **
R	1.0000	1.8371	1.8371	3777.5700	0.0000 **
V × R	3.0000	0.1629	0.0543	111.6600	0.0000 **
Error	14.0000	0.0068	0.0005		
Total	21.0000	4.0468			

** = Highly significant.

**Table 7:** Individual mean values comparison for Micronaire.

Cotton Varieties (V)	Cotton Regions (R)
V_1_ = 4.57 B	
V_2_ = 4.58 B	R_1_ = 4.84 A
V_3_ = 4.96 A	R_2_ = 4.29 B
V_4_ = 4.14 C	

Mean values with various characters varied by 0.05 probability.

**Table 8:** ANOVA Table for Cellulose (%).

Source	DF	SS	MS	F	P
V	3.0000	181.0800	60.3593	1200.5500	0.0000 **
R	1.0000	17.3100	17.3100	384.5000	0.0000 **
V × R	3.0000	198.6000	66.1900	1470.8000	0.0000 **
Error	14.0000	0.0099	0.0007		
Total	21.0000	397.6420			

** = Highly significant.

**Table 9:** Individual mean values comparison for Cellulose (%).

Cotton Varieties (V)	Cotton Regions (R)
V_1_ = 89.25 C	
V_2_ = 90.71 B	R_1_ = 89.85 B
V_3_ = 95.10 A	R_2_ = 91.55 A
V_4_ = 87.75 D	

At the 0.05 level of probability, mean values with different letters vary considerably.

**Table 10:** ANOVA table for Wax (%).

Source	DF	SS	MS	F	P
V	3.0000	0.0340	0.0110	916.0000	0.0000 **
R	1.0000	0.0037	0.0037	300.0000	0.0000 **
V × R	3.0000	0.0330	0.0110	900.0000	0.0000 **
Error	14.0000	0.0001	0.0000		
Total	21.0000	0.0708			

** = Highly significant.

**Table 11:** Individual mean values comparison for Wax (%).

Cotton Varieties (V)	Cotton Regions (R)
V_1_ = 0.59 D	
V_2_ = 0.63 B	R_1_ = 0.64 A
V_3_ = 0.60 C	R_2_ = 0.62 B
V_4_ = 0.69 A	

At the 0.05 level of probability, mean values with different letters vary considerably.

**Table 12:** ANOVA table for Ash (%).

Source	DF	SS	MS	F	P
V	3.0000	0.0220	0.0074	1772.0000	0.0000 **
R	1.0000	0.0011	0.0011	256.0000	0.0000 **
V × R	3.0000	0.0500	0.0170	4008.0000	0.0000 **
Error	14.0000	0.0003	0.0004		
Total	21.0000	0.0734			

** = Highly significant.

**Table 13:** Individual mean values comparison for Ash (%).

Cotton Varieties (V)	Cotton Regions (R)
V_1_ = 1.44 B	
V_2_ = 1.37 D	R_1_ = 1.43 A
V_3_ = 1.46 A	R_2_ = 1.41 B
V_4_ = 1.41 C	

At the 0.05 level of probability, mean values with different letters vary considerably.

**Table 14:** Correlation coefficient among different characteristics of cotton fiber.

	Fiber Length	Fiber Strength	Micronaire	Cellulose (%)	Wax (%)	Ash (%)
**Fiber Length**	1	0.683 **	−0.675 **	0.091	−0.241	−0.298
**Fiber Strength**		1	−0.021	0.539 **	−0.578 **	−0.212
**Micronaire**			1	0.317	0.028	0.433 *
**Cellulose (%)**				1	−0.529 **	0.104
**Wax (%)**					1	0.453 *
**Ash (%)**						1

**. Correlation is significant at the 0.01 level. *. Correlation is significant at the 0.05 level.

## Data Availability

The reported data is available from the corresponding authors on a reasonable request.
